# Physiochemical and Sensory Properties of a Turmeric, Ginger, and Pineapple Functional Beverage with Effects of Pulp Content

**DOI:** 10.3390/foods13050718

**Published:** 2024-02-27

**Authors:** Zahra Yusufali, Peter Follett, Marisa Wall, Xiuxiu Sun

**Affiliations:** United States Department of Agriculture, Agricultural Research Service, Daniel K. Inouye U.S. Pacific Basin Agricultural Research Center, 64 Nowelo Street, Hilo, HI 96720, USA; zahra.yusufali@usda.gov (Z.Y.); peter.follett@usda.gov (P.F.); marisa.wall@usda.gov (M.W.)

**Keywords:** *Curcuma longa*, *Zingiber officinale*, antioxidant, volatiles, sensory

## Abstract

Beverage mixtures based on pineapple juice (80–100%), with varying concentrations of turmeric (0–20%) and ginger (0–20%) juice were developed. The pineapple juice alone exhibited a total soluble solid (TSS) content of 15.90–16.03 °Brix. The total polyphenols content (TPC) varied between 0.32 and 1.79 mg GAE/mL, and the 1,1-diphenyl-2-picrylhydrazyl (DPPH) inhibition was between 40.56% and 86.19% and correlated with the TPC and curcumin and other curcuminoids. The formulations with a high pulp content showed a significantly higher TPC and greater DPPH inhibition than those with a low pulp content. Turmeric and ginger with a high amount of pulp had a higher abundance of volatile compounds. Significant differences were observed by the panelists in the taste and mouthfeel attributes and the low-pulp juices were associated with increased palatability due to the better mouthfeel, higher sweetness, and decreased bitterness, pepperiness, pulpiness, and spiciness. The pineapple juice mixtures with 10% turmeric juice and 10% or less ginger juice were most preferred by sensory panelists.

## 1. Introduction

A functional beverage is a non-alcoholic drink that includes non-traditional ingredients such as herbs, minerals, amino acids, vitamins, or additional raw fruit or vegetable ingredients, and is claimed to provide specific health benefits beyond those supplied by any normal food sources [1,2]. The functional beverage market has seen a significant increase in recent years. The US functional beverage market is forecasted to grow at a Compound Annual Growth Rate (CAGR) of 4.5% from 2022 to 2027, reaching $62 billion [1]. Globally, the functional beverage market size is expected to grow from USD 148.26 billion in 2023 to USD 203.42 billion by 2028, at a CAGR of 6.53% during the forecast period (2023–2028) [1]. This rise in demand is largely driven by consumers’ increasing concern for their well-being and a general wish for healthier lifestyles [3].

Turmeric (*Curcuma longa*) has been used in India for thousands of years as both a spice and medicinal herb [4]. It has been extensively studied for its phytochemical composition, revealing a diverse array of secondary metabolites such as monoterpenoids, sesquiterpenoids, diterpenoids, triterpenoids, curcuminoids, flavonoids, saccharides, steroids, fatty acids, and alkaloids [5]. So far, turmeric has been found to contain 50 curcuminoids, with curcumin, desmethoxycurcumin, and bisdemethoxycurcumin being the three primary ones [6,7]. These phytochemicals, mainly curcumin, contribute to turmeric’s various health benefits, including anticancer, antioxidative, anti-inflammatory, antimicrobial, antidiabetic, lipid-decreasing, hepatoprotective, and neuroprotective activities [5,8,9,10].

Ginger (*Zingiber officinale*), a widely used spice and Chinese herbal medicine, contains over 160 components, including volatile oils, gingerol analogues, diarylheptanoids, phenylalkanoids, sulfonates, steroids, and monoterpenoid glycosides compounds [11]. Recent studies highlight its diverse bioactive components, showing gastrointestinal-protective, anticancer, and obesity-preventive effects [11,12]. However, identifying the underlying effective compounds remains unclear [13,14]. The flavor profile of ginger is influenced by both volatile and non-volatile components. The volatile compounds in ginger contribute to its characteristic aroma. These compounds are primarily terpenes, which account for more than 75% of the volatile compounds present in ginger [15,16,17]. The dominant constituents among these terpenes are zingiberene, β-sesquiphellandrene, (*E*, *E*)-α-farnesene, and β-bisabolene [18]. The relative content of these compounds can vary based on variety, growing location, and various processing methods [17,19,20].

Pineapple (*Ananas comosus*) is one of the most consumed tropical fruits worldwide. It is considered a functional fruit because it is a good source of dietary fiber and other phytochemicals such as carotenoids, flavonoids, and phenolic acids [21]. Pineapple juice consumption is rising due to its refreshing flavor profile and growing awareness about its health benefits, including aiding digestion, boosting the immune system, and promoting skin health [22]. In addition to its great taste and health benefits, pineapple juice can also mask bitterness and other unfavorable taste qualities [23]. Since turmeric can have a bitter taste and ginger can have a spicy tone that might not be preferable to all consumers, pineapple juice would make an excellent candidate that can be utilized for its flavor-masking capabilities.

While prior research has examined beverages containing pineapple, turmeric, and ginger, they have not explored leveraging the masking effect of pineapple juice. Additionally, they did not investigate how varying pulp content affects the sensory and nutritional attributes of ginger and turmeric. Therefore, the purpose of this research is to evaluate formulations with varying pulp content for a functional beverage that utilizes the flavor-masking effect of pineapple juice to deliver the potential health benefits of turmeric and ginger. 

## 2. Materials and Methods

### 2.1. Juice Samples

**Figure 1:**
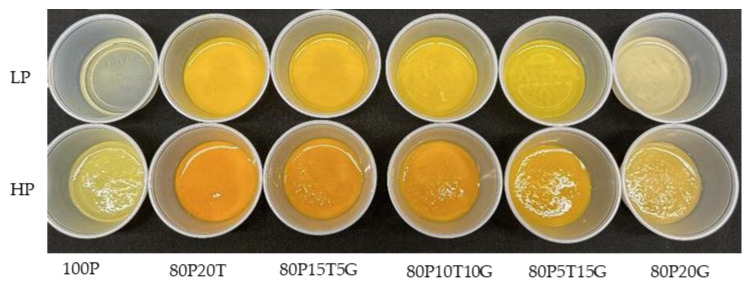
The appearance of the pineapple, turmeric, and ginger functional beverages.

The turmeric and ginger juices were supplied by Crown Pacific International (Hilo, HI, USA). Pineapple juice puree was obtained from Maui Fruit Jewels (Wailuku, HI, USA). The pineapple juice used for the control was made by juicing a fresh pineapple purchased from a local store. The formulations with either turmeric and/or ginger were created using the pineapple puree. A centrifuge operating at 15,344× *g* (10,000 rpm) for 15 min was used to produce low-pulp juice (Avanti J-26 XPI, Beckman Coulter, Indianapolis, IN, USA). Each blend was prepared to a total sample weight of 200 g. The formulations and appearance for these juices are shown in Table 1 and Figure 1, respectively. All juice blends were pasteurized at 71 °C for 30 s.

### 2.2. Total Soluble Solids (TSS) and Titratable Acidity (TA)

The total soluble solids content was measured using a digital refractometer (PAL-3, ATAGO U.S.A., Inc., Bellevue, WA, USA) and expressed as °Brix. Titratable acidity was measured using a digital fruit acidity meter (GMK-835F, G-WON, Seoul, Republic of Korea), and expressed as %. 

### 2.3. Total Polyphenols Content (TPC)

The Folin–Ciocalteu reagent assay was used to measure the total polyphenols content (TPC) [24]. For the extraction of phenolics, an aliquot of 200 µL of the juice sample was combined with 800 µL of acetone, and then centrifuged using a minicentrifuge (Minispin 22331 Hamburg, Eppendorf, Enfield, CT, USA). Following this, 25 µL aliquots of each sample or standard mixture (gallic acid) were combined with 0.25 mL of DI water, 0.75 mL of 0.2 N Folin–Ciocalteu, and 0.5 mL of a 20% sodium carbonate solution. After a brief vortexing of the mixture, 250 µL of each sample or standard was transferred into a 96-well microplate. The plate was then covered and incubated in the dark at room temperature for 2 h. Finally, the absorbance was measured at 765 nm using a microplate reader (SpecraMax M2, Molecular Devices, LLC, San Jose, CA, USA).

Quantification of phenolic content was performed using a gallic acid standard curve. Five known concentrations of gallic acid (0.1, 0.2, 0.3, 0.4, and 0.5 mg/mL) and a blank were used to obtain a standard curve (r^2^ ≥ 0.9989) and the results were expressed as mg gallic acid equivalence (GAE)/mL.

### 2.4. DPPH (1,1-diphenyl-2-picrylhydrazyl) Assay

The DPPH assay assessed juice sample DPPH radical inhibition (antioxidant capacity) using a 0.8 mM DPPH working solution, prepared by dissolving DPPH powder in 100% ethanol [25]. Each juice sample (25 mg/mL) was centrifuged after dilution (250 mg with ethanol). For the assay, 250 µL of each of the following in triplicates were added to a 96-well plate: ethanol as blank, diluted juice sample for background, 0.8 mM DPPH for control, and 100 µL of diluted sample plus 150 µL of 0.8 mM DPPH. After two hours of incubation at room temperature, the absorbance was read at 515 nm using a microplate reader (SpecraMax M2, Molecular Devices, LLC, San Jose, CA, USA). The percentage scavenging capacity was obtained using the following equation:

$$Scavenging\;effect\left(\%\right)=\frac{\left[{Abs}_{control}-\left({Abs}_{sample}-{Abs}_{sample\;background}\right)\right]}{{Abs}_{control}}\times 100$$

### 2.5. Phenolic Compounds

Three phenolic compounds (curcumin, desmethoxycurcumin, and bisdemethoxycurcumin) were analyzed using high-performance liquid chromatography with a diode array detector (HPLC-DAD) [26,27]. In the extraction of curcuminoids, 0.8 mL of juice sample was mixed with 10 mL of methanol, then centrifuged at 4000 rpm for 10 min. The resulting supernatant (0.2 mL) was filtered through a 0.2 µm membrane filter into 2 mL vials for HPLC analysis. 

The HPLC-DAD system (Agilent 1200 Quaternary HPLC, Agilent Technologies, Santa Clara, CA, USA) was equipped with a Zorbax C18 column (4.6 × 150 mm, 5 µm, Agilent Technologies, Palo Alto, CA, USA). The mobile phases consisted of acetonitrile and water with 0.1% formic acid. For the analysis of curcuminoids, the mobile phase composition was as follows, with a flow rate of 1 mL/min: isocratic 0 to 11 min of 45% acetonitrile and 55% water, 11 to 12 min 45% to 100% acetonitrile, 12 to 18 min 100% to 45% acetonitrile for eluting other compounds and cleaning the column, 18 to 23 min at 45% acetonitrile to condition the column back to the initial state for the next sample [27].

Compounds were identified by matching their retention time with standards. The quantification of curcuminoids was achieved by injecting 7 known concentrations (2.5, 5, 10, 20, 40, 80, and 160 ppm) of curcumin standard (81.2% curcumin, 16.3% desmethoxycurcumin, 1.97% bisdesmethoxycurcumin, Supelco PHR2209, Sigma Aldrich, Laramie, WY, USA) to establish two calibration curves, one for curcumin (r^2^ ≥ 0.9998) and the other for total curcuminoids (r^2^ ≥ 0.9998). 

### 2.6. Volatiles

Volatiles were examined via static headspace gas chromatography–mass spectrometry (GC-MS), following the method outlined in our prior research, with adjustments [28]. For sample preparation, 3 mL of each juice blend was combined with 1 g of NaCl in 20 mL headspace vials sealed with a silicone septum crimp cap. Headspace conditions were modified (oven temperature at 45 °C), and additional instrumentation details are described in [28]. Compound confirmation involved matching mass spectra with NIST library entries (≥90% match). Standards for 2-heptanol, 3-carene, α-phellandrene, α-pinene, eucalyptol, p-cymene, γ-terpinene, terpinolene, and turmerone were used for validation. 

### 2.7. Sensory Evaluation

Ten trained panelists assessed the juice blends as outlined in Table 1. The panelists evaluated aroma (grassy, cooked vegetable, earthy, fresh, peppery) and taste/mouth feel (sweet, sour, bitter, spicy, pulpy, and watery) descriptors on a 0–10 intensity scale, where 0 signifies none and 10 indicates the highest [28]. Each panelist received 10 mL of each juice formulation and the pineapple juice control in 50 mL plastic cups with lids (SOLO, Urbana, IL, USA). Additionally, panelists were provided water and instructed to cleanse their palates between samples.

### 2.8. Statistical Analysis

Analysis of variance (ANOVA) with Tukey’s HSD was conducted to assess the impact of formulations on juice quality attributes. Two-way ANOVA was employed to examine the effects of pulp and the interaction between blend and pulp. A correlation plot was created to evaluate the correlation between the total polyphenols, DPPH inhibition, and the curcuminoids. Additionally, a spider plot was created to visualize sensory attributes and hierarchical clustering was used to group volatiles into clusters. Principal component analysis (PCA) with unstandardized method was used to visualize the relationship between sensory and physiochemical measurements and the juice formulations. The ANOVAs, hierarchical clustering, and PCA were performed using JMP statistical analysis software (version 16; SAS Institute, Cary, NC, USA). The Pearson correlation plot was created with the RStudio and corrplot package (R version 4.2.1, R Foundation for Statistical Computing, Vienna, Austria; corrplot version 0.92).

## 3. Results and Discussion

### 3.1. Total Soluble Solids (TSS) and Titratable Acidity (TA)

The total soluble solids (TSS) and titratable acidity (TA) are shown in Table 2. The high-pulp and low-pulp 100% pineapple juices exhibited the highest TSS content at 16.03 and 15.90 °Brix, respectively, and the TSS content decreased in the formulations with 80% pineapple juice. Statistically, the TSS content was affected by the blend, pulp, and their interaction. The titratable acidity (TA) showed minimal variation between the high- and low-pulp blends (Table 2). Only the blend had a significant effect on the TA, and the sensory analysis intensity scores indicated no significant difference in the perceived sourness. Ogori et al. [29] reported a °Brix of 9.32 and TA of 0.84 for their 80P10T10G juice, which differed from our study’s same composition formulations. However, the increase in the TSS and TA in pineapple juice may correlate with changes in the citric acid content, which can be influenced by variety, maturity, storage temperature, and post-harvest handling [30,31].

### 3.2. Antioxidant Activities

**Figure 2:**
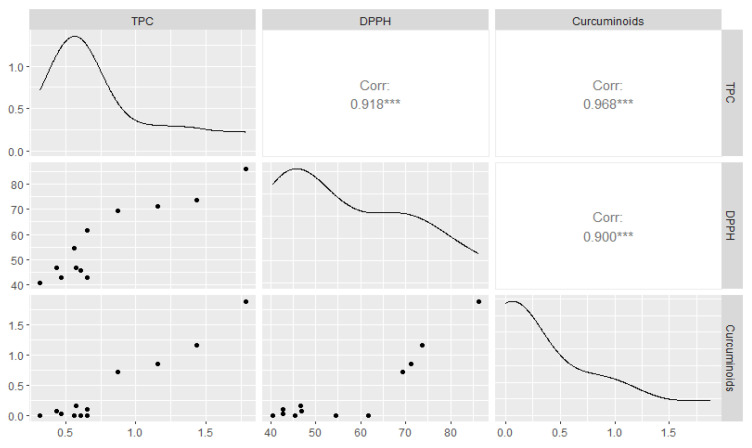
The correlation between total polyphenols content (TPC), DPPH, and curcuminoids of the turmeric, ginger, and pineapple functional beverages. The plot indicates the Pearson correlation coefficient and density plots. Each axis indicates the corresponding measurement for that analysis, i.e., mg GAE/mL for TPC, % inhibition for DPPH, and mg/mL for curcuminoids. *** indicates significance at *p* < 0.001.

Three separate analyses were conducted to identify the antioxidant components in various juices. The high-pulp juice formulations with turmeric and ginger had a higher total polyphenols content (TPC) and DPPH inhibition compared to the high-pulp pineapple control. However, removing the pulp significantly affected the TPC and DPPH inhibition compared to the low-pulp pineapple control. The TPC varied between 0.32 and 1.79 mg GAE/mL (Table 2). The juice with the lowest TPC was the 80P20G LP, while the 80P20T HP juice had the highest. The TPC decreased as the turmeric content decreased. Interestingly, the pulp content significantly influenced the TPC, with juices having a high pulp content showing twice the TPC of those with a low pulp content. The DPPH inhibition mirrored the TPC trend and a high pulp content in the juices resulted in higher DPPH inhibition compared with those with a low pulp content. Sun et al. [28] demonstrated a trend suggesting that DPPH inhibition increased with higher turmeric content, with the 20% turmeric blend exhibiting the highest inhibition. The 80P20T HP juice exhibited the highest inhibition effect at 86.19%, while the 80P20G LP juice showed the lowest at 40.56% (Table 2). As expected, the curcumin and total curcuminoid content followed a similar trend, with high-pulp juices showing significantly higher levels than low-pulp juices. The 80P20T HP juice had the highest content of curcumin and total curcuminoids (Table 2). Since curcuminoids are exclusive to turmeric, they were not detected in the control pineapple juices and the ginger-only blends. The main curcuminoids in turmeric, namely curcumin and desmethoxycurcumin, have exhibited the most significant antioxidant activity [6]. A significant positive correlation was observed among these three measurements (Figure 2). The correlation coefficients were as follows: TPC and DPPH inhibition at 0.918, TPC and curcuminoids at 0.968, and DPPH inhibition and curcuminoids at 0.90. Therefore, the major antioxidant activity and total phenolics were dependent on the curcuminoid content, which in turn was dependent on the turmeric content of the juices.

### 3.3. Volatiles

**Figure 3:**
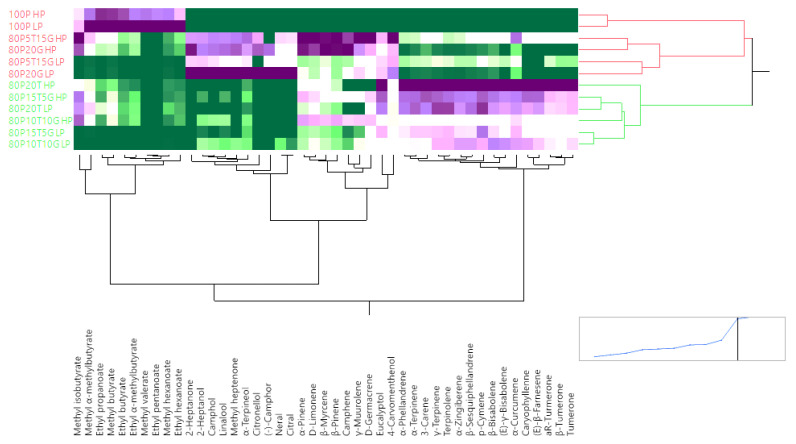
The hierarchical cluster dendrogram of the volatile compounds in the turmeric, ginger, and pineapple functional beverages. Green signifies the lack of the volatile compound in the formulation, while purple indicates the presence of the compound. The deeper the green, the greater the absence of the compound. The darker the purple, the higher the abundance of the compound, and vice versa.

The juice samples analyzed in this study revealed a variety of volatile compounds, which are detailed in Table 3, arranged by elution order. In total, 45 volatile compounds were identified across the juices. Among these, ten volatiles were identified in pineapple that were esters. Other volatile compounds such as aldehydes, ketones, lactones, and alcohols also have been identified in pineapple [32,33]. In the formulations of pineapple juice with turmeric and ginger, a noticeable decrease in the intensity of these ester volatiles was observed, particularly in the 20% turmeric high-pulp blend (80P20T HP). Interestingly, reducing the turmeric content resulted in a slight increase in these ten ester volatiles. Methyl hexanoate stood out as the most intense volatile detected in pineapple juice, imparting a fruity, fresh, and sweet aroma. In the hierarchical cluster dendrogram (Figure 3), the ten ester volatiles formed a distinct cluster for pineapple juice. Ginger and turmeric, both members of the *Zingiberaceae* family, share many volatile aromas [17,29]. Common volatiles to both include terpenes such as 3-carene, α-terpinene, α-zingiberene, and β-sesquiphellandrene. These shared compounds formed distinct clusters in the dendrogram (Figure 3). Unique to turmeric are the turmerones, including ar-turmerone, turmerone, and β-turmerone [32]. The volatile oil from turmeric rhizomes also contains eucalyptol (11.2%), α-turmerone (11.1%), β-caryophyllene (9.8%), ar-turmerone (7.3%), and β-sesquiphellandrene (7.1%) [34]. These volatiles formed a separate cluster in the dendrogram (Figure 3).

The ginger-specific compounds included 2-heptanone, 2-heptanol, camphene, linalool, (-)-camphor, camphol, citronellol, neral, citral, and D-germacrene. None of these volatiles were found in the 80P20T blends, indicating their absence in turmeric. The main volatile compounds in ginger are mono- and sesquiterpenes and are responsible for the fresh, citrus-like, minty, spicy, and floral odor notes of ginger [15]. Various methods have been employed to extract volatile compounds from ginger samples, both peeled and unpeeled. These methods encompass static headspace (SHA), solid-phase microextraction (SPME), solvent-assisted flavor evaporation (SAFE), and direct immersion-stir bar sorptive extraction (DI-SBSE). SHA has been used in other studies to identify ten terpenes, two aldehydes, and one alcohol. However, when compared to other extraction techniques, SHA yields the fewest number of volatile compounds in ginger [15,18]. As this study utilized a static headspace sampling method, certain distinctive ginger volatile compounds may have gone undetected, potentially compromising their association with the sensory perception of ginger in these juice formulations. 

Most volatiles in the juice samples varied due to blend, pulp, or blend–pulp factors. Turmeric-associated volatiles like, ar-turmerone, turmerone, and β-turmerone showed no variation due to any factors (Table 3). Ten pineapple-associated volatiles were more abundant in low-pulp juice, contrasting with blends where a high amount of pulp had more abundance in some volatiles. This suggests that turmeric and ginger with pulp has a higher abundance of volatile compounds.

### 3.4. Sensory Evaluation

**Figure 4:**
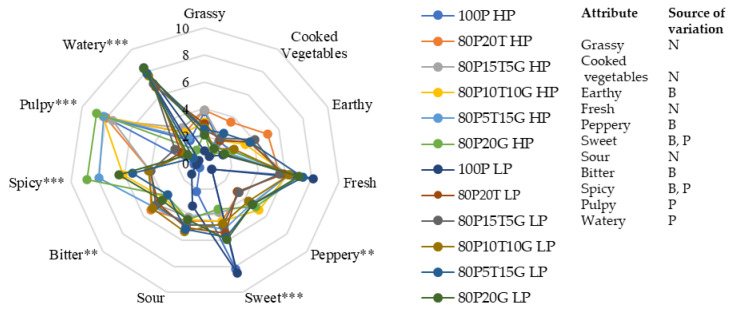
The sensory profile and the attribute’s source of variation in the turmeric, ginger, and pineapple functional beverages (the asterisks on the top right of attributes refer to statistical significance using Fisher’s LSD multiple comparison test (**: *p* < 0.01, ***: *p* < 0.001); Source of variation by two-way ANOVA: N: no effect; B: blend; P: pulp).

The sensory profile of each juice blend is depicted in the spider plot chart (Figure 4). To differentiate between the low-pulp and high-pulp blends, we included ‘watery’ and ‘pulpy’ in the mouthfeel descriptors. The panelists were able to distinguish between these blends based on the results. Significant differences were observed in the taste and mouthfeel attributes, specifically in terms of bitterness, pepperiness, pulpiness, spiciness, and sweetness. The blend 80P20T HP scored highest for bitterness, while the 80P20G HP blend had the highest spiciness score of 8.6. Its low-pulp counterpart scored 2 points less in spiciness, but it was still considered high. No significant differences were found in the rest of the descriptors. The pulp content in the juices primarily affected the descriptors of the sweetness, spiciness, wateriness, and pulpiness. 

**Figure 5:**
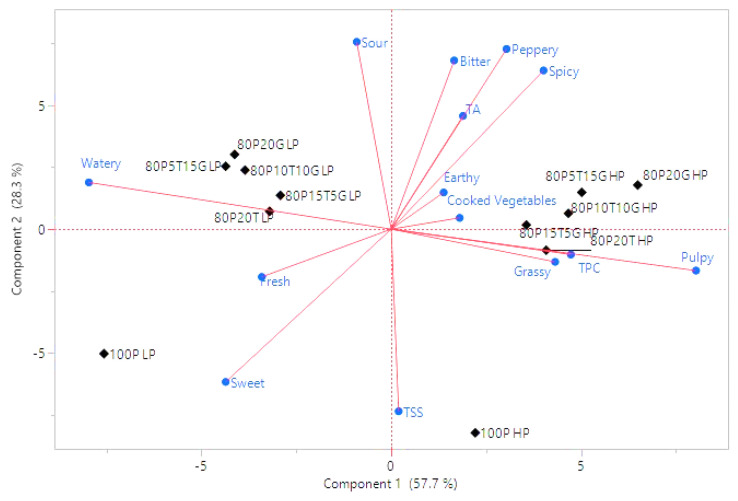
The principal component analysis (PCA) plot of sensory descriptors for the turmeric, ginger, and pineapple functional beverage. Blue indicates sensory attributes and chemical measurements and black indicates samples.

In the principal component analysis (PCA), the first two components accounted for 86% of the variation, with PC1 contributing 57.7% and PC2 contributing 28.3% (Figure 5). Notably, PC1 separated the low-pulp juices on the left side (correlating with the ‘watery’ description) and the high-pulp juices on the right side (correlating with the ‘pulpy’ description). Most of the flavor descriptors were associated with the high-pulp juices (Figure 5). The descriptors of sweetness, freshness, and the TSS content were aligned with the control pineapple juices, as they separated on the lower section of PC2 (Figure 5). The 15% and 20% ginger high-pulp juice samples showed a significantly higher score for spicy than the other juice samples, which was probably due to the higher mono- and sesquiterpenes in ginger [15]. The 20% turmeric juice samples had a significantly higher score for bitterness, which is positively associated with the bitter phenolic compounds in turmeric [28]. Similar to previous research with winter melon, the low-pulp juice showed the highest acceptability from the sensory panel [35].

## 4. Conclusions

This study offers an overview of the various pineapple juice blends that can be created with ginger and turmeric, serving as functional beverages. These are choices consumers may make, given their health and nutritional benefits. The masking effect of pineapple juice enables the inclusion of a higher content of ginger and turmeric in the blends, enhancing the quality of the functional beverage. While the high-pulp blends demonstrated a superior nutritional quality, they were associated with descriptors such as bitterness and spiciness, which may not appeal to all consumers. On the other hand, the low-pulp blends, although less associated with negatively connotated descriptors, still offer numerous nutritional benefits, and could serve as viable options for functional drinks, particularly the 80P10T10G LP blend. Future research directions could explore alternative processing methods beyond pasteurization, as the application of heat may modify the bioactivity of compounds. Processes such as high-pressure pasteurization, which does not rely on heat, could present a promising avenue for enhancing the nutritional quality of the functional beverage formulations. Overall, the incorporation of Hawaiian-grown organic turmeric and ginger not only enhances the quality of the blends but also benefits the local farmers and distributors of these commodities.

## Figures and Tables

**Table 1:** The formulations and abbreviations of the turmeric, ginger, and pineapple functional beverages.

Juice Formulation	Abbreviation
100% Pineapple, High Pulp (Control)	100P HP
80% Pineapple 20% Turmeric, High Pulp	80P20T HP
80% Pineapple 15% Turmeric 5% Ginger, High Pulp	80P15T5G HP
80% Pineapple 10% Turmeric 10% Ginger, High Pulp	80P10T10G HP
80% Pineapple 5% Turmeric 15% Ginger, High Pulp	80P5T15G HP
80% Pineapple 20% Ginger, High Pulp	80P20G HP
100% Pineapple, Low Pulp (Control)	100P LP
80% Pineapple 20% Turmeric, Low Pulp	80P20T LP
80% Pineapple 15% Turmeric 5% Ginger, Low Pulp	80P15T5G LP
80% Pineapple 10% Turmeric 10% Ginger, Low Pulp	80P10T10G LP
80% Pineapple 5% Turmeric 15% Ginger, Low Pulp	80P5T15G LP
80% Pineapple 20% Ginger, Low Pulp	80P20G LP

**Table 2:** The total soluble solids (TSS), titratable acidity (TA), total polyphenols content (TPC), % DPPH inhibition, curcumin, and total curcuminoids of the turmeric, ginger, and pineapple functional beverage.

Samples	TSS (°Brix)	TA (%)	Total Polyphenols (mg GAE/mL)	DPPH Assay (%DPPH)	Curcumin (mg/mL)	Total Curcuminoids (mg/mL)
100P HP	15.90 ± 0.00 a	0.57 ± 0.01 c	0.61 ± 0.105 ef	45.54 ± 2.94 ef	ND f	ND e
80P20T HP	14.60 ± 0.17 b	0.66 ± 0.02 ab	1.79 ± 0.109 a	86.19 ± 3.32 a	1.11 ± 0.050 a	1.88 ± 0.108 a
80P15T5G HP	13.13 ± 0.06 de	0.65 ± 0.04 ab	1.43 ± 0.023 b	73.63 ± 8.05 b	0.67 ± 0.056 b	1.15 ± 0.088 b
80P10T10G HP	13.93 ± 0.06 c	0.67 ± 0.03 a	1.15 ± 0.051 c	71.30 ± 3.75 bc	0.51 ± 0.030 c	0.86 ± 0.057 c
80P5T15G HP	13.90 ± 0.00 c	0.65 ± 0.06 ab	0.87 ± 0.035 d	69.30 ± 2.21 bc	0.40 ± 0.019 d	0.73 ± 0.007 c
80P20G HP	13.83 ± 0.06 c	0.59 ± 0.02 bc	0.56 ± 0.016 efg	54.61 ± 0.85 de	ND f	ND e
100P LP	16.03 ± 0.25 a	0.57 ± 0.01 c	0.65 ± 0.031 e	61.80 ± 5.87 cd	ND f	ND e
80P20T LP	14.53 ± 0.06 b	0.66 ± 0.01 ab	0.57 ± 0.039 efg	46.65 ± 1.18 ef	0.09 ± 0.010 e	0.16 ± 0.018 d
80P15T5G LP	13.17 ± 0.06 d	0.66 ± 0.01 ab	0.65 ± 0.016 e	42.71 ± 1.15 f	0.06 ± 0.010 e	0.11 ± 0.020 d
80P10T10G LP	12.93 ± 0.06 def	0.64 ± 0.01 abc	0.43 ± 0.030 gh	46.83 ± 2.56 ef	0.04 ± 0.005 e	0.08 ± 0.010 d
80P5T15G LP	12.87 ± 0.06 ef	0.63 ± 0.03 abc	0.47 ± 0.035 fgh	42.68 ± 1.35 f	0.02 ± 0.003 e	0.04 ± 0.007 d
80P20G LP	12.70 ± 0.00 f	0.60 ± 0.01 abc	0.32 ± 0.013 h	40.56 ± 0.35 f	ND f	ND e
Source of Variation	B, P, B*P	B	B, P, B*P	B, P, B*P	B, P, B*P	B, P, B*P

Mean values ± standard deviations followed by different letters within a column indicate significant differences using a Tukey’s HSD test at α = 0.05. Source of variation by two-way ANOVA: B: blend and P: pulp as main effects; B*P: blend*pulp the interaction between them. ND: not detected.

**Table 3:** The volatile profile of the turmeric, ginger, and pineapple functional beverages.

			Average Abundance (Total Ion Current × 10^6^)												
			High Pulp						Low Pulp						Source of Variation
Peak #	Compounds	Ret. Time	100P	80P20T	80P15T5G	80P10T10G	80P5T15G	80P20G	100P	80P20T	80P15T5G	80P10T10G	80P5T15G	80P20G	
1	Methyl isobutyrate	2.894	0.10 ± 0.01 ade	0.08 ± 0.02 ef	0.13 ± 0.00 bcd	0.14 ± 0.00 abc	0.16 ± 0.01 a	0.14 ± 0.01 ab	0.11 ± 0.00 cde	0.12 ± 0.01 bcd	0.00 ± 0.00 g	0.00 ± 0.00 g	0.00 ± 0.00 g	0.00 ± 0.00 g	B*P
2	Ethyl propanoate	3.205	1.03 ± 0.02 b	0.09 ± 0.04 de	0.07 ± 0.00 e	0.06 ± 0.01 e	0.25 ± 0.02 c	0.17 ± 0.03 cd	1.14 ± 0.06 a	0.18 ± 0.02 c	0.00 ± 0.00 e	0.00 ± 0.00 e	0.00 ± 0.00 e	0.00 ± 0.00 e	B
3	Methyl butyrate	3.334	5.90 ± 0.10 b	1.02 ± 0.11 e	1.47 ± 0.13 cd	1.48 ± 0.02 cd	1.83 ± 0.08 c	1.40 ± 0.13 de	6.62 ± 0.37 a	1.54 ± 0.15 cd	0.26 ± 0.01 f	0.26 ± 0.01 f	0.31 ± 0.02 f	0.34 ± 0.02 f	B, P, B*P
4	Methyl α-methylbutyrate	4.158	4.66 ± 0.04 b	2.07 ± 0.16 f	3.06 ± 0.09 e	3.07 ± 0.03 de	3.59 ± 0.09 c	3.13 ± 0.06 de	5.99 ± 0.39 a	3.50 ± 0.26 cd	0.45 ± 0.03 g	0.43 ± 0.03 g	0.49 ± 0.00 g	0.53 ± 0.01 g	B, P, B*P
5	Ethyl butyrate	4.549	2.22 ± 0.10 b	0.11 ± 0.05 cd	0.14 ± 0.02 cd	0.16 ± 0.02 cd	0.27 ± 0.02 c	0.16 ± 0.01 cd	2.77 ± 0.18 a	0.18 ± 0.04 cd	0.00 ± 0.00 cd	0.00 ± 0.00 cd	0.00 ± 0.00 d	0.00 ± 0.00 d	B, B*P
6	Methyl valerate	4.941	0.15 ± 0.01 a	0.00 ± 0.00 c	0.00 ± 0.00 c	0.00 ± 0.00 c	0.00 ± 0.00 c	0.00 ± 0.00 c	0.22 ± 0.02 b	0.00 ± 0.00 c	0.00 ± 0.00 c	0.00 ± 0.00 c	0.00 ± 0.00 c	0.00 ± 0.00 c	P*
7	Ethyl α-methylbutyrate	5.395	1.19 ± 0.02 b	0.05 ± 0.01 c	0.13 ± 0.01 c	0.16 ± 0.02 c	0.21 ± 0.01 c	0.19 ± 0.01 c	1.86 ± 0.22 a	0.15 ± 0.02 c	0.00 ± 0.00 c	0.00 ± 0.00 c	0.00 ± 0.00 c	0.00 ± 0.00 c	B, B*P
8	2-Heptanone	6.105	0.00 ± 0.00 c	0.00 ± 0.00 c	0.00 ± 0.00 c	0.00 ± 0.00 c	0.07 ± 0.00 b	0.11 ± 0.02 a	0.00 ± 0.00 c	0.00 ± 0.00 c	0.00 ± 0.00 c	0.00 ± 0.00 c	0.05 ± 0.01 b	0.11 ± 0.01 a	B
9	2-Heptanol	6.265	0.00 ± 0.00 d	0.00 ± 0.00 d	0.20 ± 0.05 c	0.39 ± 0.09 c	1.77 ± 0.05 b	2.00 ± 0.85 b	0.00 ± 0.00 d	0.00 ± 0.00 d	0.07 ± 0.02 c	0.46 ± 0.04 c	1.39 ± 0.04 b	2.94 ± 0.15 a	B, B*P
10	Ethyl pentanoate	6.279	0.08 ± 0.00 b	0.00 ± 0.00 c	0.00 ± 0.00 c	0.00 ± 0.00 c	0.00 ± 0.00 c	0.00 ± 0.00 c	0.13 ± 0.01 a	0.00 ± 0.00 c	0.00 ± 0.00 c	0.00 ± 0.00 c	0.00 ± 0.00 c	0.00 ± 0.00 c	P
11	Methyl hexanoate	6.688	10.60 ± 0.71 b	0.58 ± 0.05 c	0.49 ± 0.08 c	0.48 ± 0.08 c	0.75 ± 0.05 c	0.59 ± 0.08 c	16.64 ± 1.33 a	0.98 ± 0.11 c	0.05 ± 0.00 c	0.07 ± 0.01 c	0.08 ± 0.01 c	0.11 ± 0.01 c	B, P, B*P
12	α-Pinene	6.853	0.00 ± 0.00 f	2.13 ± 0.58 cde	2.76 ± 0.27 cd	2.96 ± 0.20 bc	4.42 ± 0.44 a	4.29 ± 1.22 ab	0.00 ± 0.00 f	1.38 ± 0.24 e	1.09 ± 0.09 e	1.22 ± 0.09 e	1.09 ± 0.08 e	1.49 ± 0.11 de	B, P, B*P
13	Camphene	7.118	0.00 ± 0.00 e	0.00 ± 0.00 e	3.00 ± 0.31 bcd	5.92 ± 0.43 b	11.08 ± 0.69 a	13.03 ± 2.89 a	0.00 ± 0.00 e	0.00 ± 0.00 e	0.41 ± 0.04 d	1.17 ± 0.06 d	2.06 ± 0.14 cd	4.39 ± 0.33 bc	B, P, B*P
14	β-Pinene	7.609	0.00 ± 0.00 e	0.16 ± 0.04 cd	0.29 ± 0.02 bc	0.39 ± 0.03 b	0.64 ± 0.04 a	0.63 ± 0.16 a	0.00 ± 0.00 e	0.11 ± 0.02 d	0.09 ± 0.01 d	0.12 ± 0.01 d	0.13 ± 0.01 d	0.13 ± 0.01 d	B, P, B*P
15	Methyl heptenone	7.774	0.00 ± 0.00 e	0.00 ± 0.00 e	0.00 ± 0.00 e	0.00 ± 0.00 e	0.26 ± 0.01 abc	0.30 ± 0.12 ab	0.00 ± 0.00 e	0.00 ± 0.00 e	0.00 ± 0.00 e	0.05 ± 0.01 d	0.12 ± 0.01 cd	0.38 ± 0.03 a	B, B*P
16	β-Myrcene	7.827	0.00 ± 0.00 e	0.87 ± 0.25 bcd	1.21 ± 0.16 bc	1.37 ± 0.12 b	2.22 ± 0.19 a	2.26 ± 0.61 a	0.00 ± 0.00 e	0.64 ± 0.14 cd	0.53 ± 0.04 d	0.66 ± 0.05 cd	0.72 ± 0.06 bcd	1.06 ± 0.07 bcd	B, P, B*P
17	Ethyl hexanoate	7.970	1.91 ± 0.03 b	0.10 ± 0.04 c	0.16 ± 0.04 c	0.20 ± 0.01 c	0.33 ± 0.01 c	0.22 ± 0.04 c	4.64 ± 0.35 a	0.19 ± 0.08 c	0.00 ± 0.00 c	0.00 ± 0.00 c	0.00 ± 0.00 c	0.00 ± 0.00 c	B, B*P
18	α-Phellandrene	8.070	0.00 ± 0.00 g	40.15 ± 8.28 a	30.81 ± 3.16 ab	16.15 ± 1.31 cd	9.74 ± 0.87 de	0.41 ± 0.12 ef	0.00 ± 0.00 g	29.79 ± 4.59 b	20.17 ± 1.88 c	17.73 ± 0.76 cd	8.51 ± 0.66 def	0.18 ± 0.03 f	B, P, B*P
19	3-Carene	8.171	0.00 ± 0.00 h	0.87 ± 0.17 a	0.74 ± 0.06 ab	0.47 ± 0.02 cd	0.35 ± 0.03 de	0.11 ± 0.03 fg	0.00 ± 0.00 h	0.65 ± 0.08 bc	0.51 ± 0.05 cd	0.44 ± 0.02 de	0.26 ± 0.02 ef	0.04 ± 0.00 g	B, P, B*P
20	α-Terpinene	8.278	0.00 ± 0.00 g	1.27 ± 0.27 a	1.00 ± 0.10 ab	0.55 ± 0.04 cd	0.35 ± 0.02 de	0.06 ± 0.01 ef	0.00 ± 0.00 g	1.03 ± 0.15 ab	0.72 ± 0.06 bc	0.66 ± 0.03 cd	0.35 ± 0.02 def	0.04 ± 0.00 f	B, P, B*P
21	p-Cymene	8.411	0.00 ± 0.00 f	1.42 ± 0.32 a	1.20 ± 0.15 ab	0.78 ± 0.02 cd	0.69 ± 0.04 d	0.11 ± 0.01 e	0.00 ± 0.00 f	1.31 ± 0.13 ab	1.14 ± 0.10 abc	0.97 ± 0.06 bcd	0.65 ± 0.04 d	0.07 ± 0.00 e	B
22	D-Limonene	8.483	0.00 ± 0.00 f	2.92 ± 0.67 cde	3.80 ± 0.44 cd	4.25 ± 0.36 bc	6.76 ± 0.63 a	5.99 ± 1.81 ab	0.00 ± 0.00 f	2.31 ± 0.35 cde	1.82 ± 0.18 e	2.20 ± 0.11 de	1.77 ± 0.15 e	1.83 ± 0.17 de	B, P, B*P
23	Eucalyptol	8.537	0.00 ± 0.00 e	48.01 ± 6.98 a	42.98 ± 3.22 ab	32.99 ± 1.27 cd	36.83 ± 1.59 bc	28.25 ± 3.34 d	0.00 ± 0.00 e	40.78 ± 1.95 abc	36.75 ± 1.02 bc	33.12 ± 1.45 cd	35.23 ± 0.13 bcd	35.82 ± 0.43 bcd	B, B*P
24	γ-Terpinene	8.979	0.00 ± 0.00 f	1.16 ± 0.25 a	0.96 ± 0.10 ab	0.56 ± 0.05 cd	0.41 ± 0.02 d	0.05 ± 0.01 e	0.00 ± 0.00 f	1.04 ± 0.14 a	0.71 ± 0.06 bc	0.73 ± 0.04 bc	0.38 ± 0.02 d	0.03 ± 0.00 e	B, B*P
25	Terpinolene	9.464	0.00 ± 0.00 f	11.05 ± 2.51 a	8.67 ± 1.04 ab	4.80 ± 0.42 cd	3.14 ± 0.27 d	0.24 ± 0.13 e	0.00 ± 0.00 f	9.79 ± 1.22 a	6.75 ± 0.63 bc	6.85 ± 0.29 bc	3.37 ± 0.27 d	0.14 ± 0.01 e	B, B*P
26	Linalool	9.643	0.00 ± 0.00 d	0.00 ± 0.00 d	0.09 ± 0.01 c	0.19 ± 0.04 c	0.83 ± 0.07 ab	0.95 ± 0.56 ab	0.00 ± 0.00 d	0.00 ± 0.00 d	0.04 ± 0.02 c	0.14 ± 0.03 c	0.50 ± 0.04 bc	1.28 ± 0.06 a	B
27	(-)-Camphor	10.407	0.00 ± 0.00 c	0.00 ± 0.00 c	0.00 ± 0.00 c	0.00 ± 0.00 c	0.00 ± 0.00 c	0.10 ± 0.04 ab	0.00 ± 0.00 c	0.00 ± 0.00 c	0.00 ± 0.00 c	0.00 ± 0.00 c	0.05 ± 0.01 bc	0.14 ± 0.01 a	B
28	Camphol	10.727	0.00 ± 0.00 d	0.00 ± 0.00 d	0.00 ± 0.00 d	0.13 ± 0.01 c	0.61 ± 0.10 ab	0.66 ± 0.28 ab	0.00 ± 0.00 d	0.00 ± 0.00 d	0.00 ± 0.00 d	0.13 ± 0.01 c	0.41 ± 0.02 bc	0.93 ± 0.07 a	B, B*P
29	4-Carvomenthenol	10.896	0.00 ± 0.00 c	0.11 ± 0.05 b	0.13 ± 0.02 b	0.09 ± 0.01 b	0.27 ± 0.04 a	0.17 ± 0.10 ab	0.00 ± 0.00 c	0.09 ± 0.02 b	0.10 ± 0.00 b	0.11 ± 0.00 b	0.17 ± 0.02 ab	0.20 ± 0.02 ab	B
30	α-Terpineol	11.092	0.00 ± 0.00 e	0.09 ± 0.05 cd	0.14 ± 0.04 cd	0.16 ± 0.01 cd	0.86 ± 0.19 ab	0.69 ± 0.41 b	0.00 ± 0.00 e	0.06 ± 0.02 d	0.12 ± 0.00 cd	0.15 ± 0.02 cd	0.51 ± 0.07 bc	1.14 ± 0.12 a	B, B*P
31	Citronellol	11.608	0.00 ± 0.00 a	0.00 ± 0.00 a	0.00 ± 0.00 a	0.00 ± 0.00 a	0.18 ± 0.11 a	0.26 ± 0.22 a	0.00 ± 0.00 a	0.00 ± 0.00 a	0.00 ± 0.00 a	0.00 ± 0.00 a	0.00 ± 0.00 a	0.34 ± 0.01 a	N
32	Neral	11.809	0.00 ± 0.00 b	0.00 ± 0.00 b	0.00 ± 0.00 b	0.00 ± 0.00 b	0.31 ± 0.04 b	0.18 ± 0.20 b	0.00 ± 0.00 b	0.00 ± 0.00 b	0.00 ± 0.00 b	0.06 ± 0.02 b	0.22 ± 0.01 b	0.83 ± 0.07 a	B, B*P
33	Citral	12.226	0.00 ± 0.00 c	0.00 ± 0.00 c	0.00 ± 0.00 c	0.00 ± 0.00 c	0.27 ± 0.07 b	0.13 ± 0.14 bc	0.00 ± 0.00 c	0.00 ± 0.00 c	0.00 ± 0.00 c	0.02 ± 0.01 c	0.18 ± 0.02 bc	0.75 ± 0.09 a	B, B*P
34	Caryophyllenne	14.371	0.00 ± 0.00 a	0.08 ± 0.04 a	0.06 ± 0.01 a	0.03 ± 0.00 a	0.03 ± 0.00 a	0.00 ± 0.00 a	0.00 ± 0.00 a	0.05 ± 0.02 a	0.03 ± 0.01 a	0.05 ± 0.02 a	0.00 ± 0.00 a	0.00 ± 0.00 a	N
35	EUR -β-Farnesene	14.712	0.00 ± 0.00 a	0.13 ± 0.08 a	0.10 ± 0.02 a	0.04 ± 0.00 a	0.04 ± 0.02 a	0.00 ± 0.00 a	0.00 ± 0.00 a	0.08 ± 0.00 a	0.06 ± 0.02 a	0.07 ± 0.03 a	0.00 ± 0.00 a	0.00 ± 0.00 a	N
36	α-Curcumene	15.094	0.00 ± 0.00 c	1.81 ± 0.85 a	1.44 ± 0.22 ab	1.16 ± 0.36 ab	1.50 ± 0.26 a	0.42 ± 0.29 b	0.00 ± 0.00 c	1.37 ± 0.37 ab	1.24 ± 0.20 ab	1.32 ± 0.27 ab	0.75 ± 0.12 ab	0.40 ± 0.02 b	B
37	D-Germacrene	15.151	0.00 ± 0.00 c	0.00 ± 0.00 c	0.17 ± 0.02 ab	0.18 ± 0.04 ab	0.31 ± 0.06 a	0.19 ± 0.12 ab	0.00 ± 0.00 c	0.10 ± 0.03 b	0.10 ± 0.02 b	0.13 ± 0.04 b	0.11 ± 0.02 b	0.13 ± 0.01 b	B*P
38	α-Zingiberene	15.245	0.00 ± 0.00 d	43.23 ± 19.38 a	31.75 ± 5.24 ab	16.32 ± 3.46 bc	11.75 ± 3.02 bc	0.98 ± 0.86 c	0.00 ± 0.00 d	29.16 ± 6.69 ab	22.61 ± 2.74 ab	28.07 ± 8.69 ab	10.31 ± 2.10 bc	0.39 ± 0.05 c	B
39	γ-Muurolene	15.366	0.00 ± 0.00 c	0.00 ± 0.00 c	0.39 ± 0.05 b	0.67 ± 0.13 ab	1.34 ± 0.28 a	0.86 ± 0.59 ab	0.00 ± 0.00 c	0.00 ± 0.00 c	0.12 ± 0.00 b	0.31 ± 0.09 b	0.36 ± 0.05 b	0.64 ± 0.04 ab	B,P
40	β-Bisabolene	15.414	0.00 ± 0.00 d	4.10 ± 1.93 a	3.23 ± 0.52 ab	2.11 ± 0.52 abc	2.07 ± 0.48 abc	0.49 ± 0.40 c	0.00 ± 0.00 d	2.90 ± 0.67 ab	2.40 ± 0.37 abc	3.09 ± 0.95 ab	1.35 ± 0.25 bc	0.34 ± 0.02 c	B
41	β-Sesquiphellandrene	15.614	0.00 ± 0.00 d	16.12 ± 7.97 a	11.81 ± 2.14 ab	6.59 ± 1.76 bc	5.38 ± 1.46 bc	0.62 ± 0.50 c	0.00 ± 0.00 d	12.59 ± 0.63 ab	8.63 ± 1.11 abc	10.66 ± 3.42 ab	4.11 ± 0.84 bc	0.43 ± 0.04 c	B
42	(E)-γ-Bisabolene	15.715	0.00 ± 0.00 c	0.59 ± 0.35 a	0.42 ± 0.06 ab	0.27 ± 0.09 ab	0.21 ± 0.07 ab	0.00 ± 0.00 c	0.00 ± 0.00 c	0.38 ± 0.08 ab	0.30 ± 0.05 ab	0.41 ± 0.12 ab	0.15 ± 0.03 b	0.00 ± 0.00 c	B
43	ar-Turmerone	17.301	0.00 ± 0.00 a	0.49 ± 0.39 a	0.27 ± 0.10 a	0.18 ± 0.05 a	0.20 ± 0.11 a	0.00 ± 0.00 a	0.00 ± 0.00 a	0.24 ± 0.09 a	0.18 ± 0.01 a	0.23 ± 0.09 a	0.11 ± 0.01 a	0.00 ± 0.00 a	N
44	Tumerone	17.351	0.00 ± 0.00 a	5.12 ± 3.99 a	2.76 ± 1.05 a	1.53 ± 0.26 a	1.37 ± 0.72 a	0.00 ± 0.00 a	0.00 ± 0.00 a	2.74 ± 0.98 a	2.01 ± 0.06 a	2.47 ± 1.26 a	0.86 ± 0.01 a	0.00 ± 0.00 a	N
45	β-Tumerone	17.729	0.00 ± 0.00 a	0.74 ± 0.57 a	0.38 ± 0.15 a	0.27 ± 0.04 a	0.24 ± 0.13 a	0.00 ± 0.00 a	0.00 ± 0.00 a	0.37 ± 0.13 a	0.29 ± 0.02 a	0.33 ± 0.16 a	0.12 ± 0.02 a	0.00 ± 0.00 a	N

Mean values ± standard deviations followed by different letters within a row indicate significant differences using a Tukey’s HSD test at α = 0.05. Source of variation by two-way ANOVA: B: blend and P: pulp as main effects; B*P: blend*pulp the interaction between them.

## Data Availability

The raw data supporting the conclusions of this article will be made available by the authors on request.
